# Substantial unexplained variation in cancer risks for MLH1 and MSH2 mutation carriers

**DOI:** 10.1186/1897-4287-10-S2-A33

**Published:** 2012-04-12

**Authors:** JG Dowty, AK Win, D Buchanan, RJ Macinnis, N Lindor, SN Thibodeau, G Casey, S Gallinger, L LeMarchand, P Newcomb, R Haile, J Goldblatt, S Parry, FA Macrae, JL Hopper, MA Jenkins

**Affiliations:** 1 School of Population Health, Uni Melbourne, Australia; 2 Familial Cancer Laboratory, Queensland Institute of Medical Research, Australia; 3 Dept. Medical Genetics, Mayo Clinic, USA; 4 Dept. Laboratory Medicine and Pathology, Mayo Clinic, USA; 5 Dept. Preventive Medicine, University of Southern California, USA; 6 Mount Sinai Hospital, University of Toronto, Canada; 7 University of Hawaii Cancer Research Center, USA; 8 Cancer Prevention Program, Fred Hutchinson Cancer Research Center, USA; 9 Genetic Services and Familial Cancer Program of Western Australia, Australia; 10 New Zealand Familial Gastrointestinal Cancer Registry, Auckland City Hospital, New Zealand; 11 Dept. Colorectal Medicine and Genetics, The Royal Melbourne Hospital, Australia

## Background

Germline mutations in the DNA mismatch repair genes *MLH1* and *MSH2* are associated with substantially increased risks of colorectal cancer (CRC), endometrial cancer (EC) and certain other cancers. Due to the rarity of these mutations, previous studies have been underpowered to provide precise estimates of risks.

## Methods

We studied 167 *MLH1* and 225 *MSH2* mutation-carrying families comprising 17,352 members from the Colon Cancer Family Registry. Probands were recruited either because they had a family history of cancer (n=274) or from cancer registries independently of family history (n=118). Hazard ratios (HRs) of cancer risks for carriers compared with the general population and age-specific cumulative risks for carriers (penetrance) were estimated using modified segregation analysis conditioned on ascertainment. Heterogeneity in risks for carriers was modelled with a polygenic risk modifier (as in the BOADICEA model).

## Results

The age-specific incidence of CRC for male *MLH1* mutation carriers was estimated to be 222 times (95% CI: 152-324) that for the population at ages 40 years and younger but only 6.1 times higher (95% CI: 2.4-15.5) after age 60 years. This decline (p<0.0001) in the CRC HR with age was also evident for female *MLH1* mutation carriers (p=0.002) but was less marked. Male *MLH1* mutation carriers on average had higher CRC HRs than female carriers (p=0.004). The estimated CRC HRs for *MSH2* mutation carriers were similar to those for *MLH1*, with any differences consistent with chance (p>0.3). The estimated EC HRs also decreased sharply with age (p=0.0004 and 0.001 for *MLH1* and *MSH2*, respectively) and no difference by gene was observed (p=0.7). There was strong evidence for an unmeasured polygenic modifier of risk (p<0.0001). The estimated average cumulative risks (95% CI) of CRC to age 70 years were 44% (35-54) for male carriers and 38% (30-48) for female carriers, and corresponding EC risks were 22% (14-31). However for carriers in the lowest vs. highest quartiles of polygenic risk (respectively) these were 4.9% vs. 93% for male CRC, 3.5% vs. 88% for female CRC and 1.5% vs. 59% for EC.

## Conclusions

This international study shows that, although the average cancer risks for *MLH1* and *MSH2* mutation carriers are similar, there is substantial unexplained variation in risks due to differences by mutation or by genetic or environmental modifiers. This finding has implications for the counselling and clinical management of mutation carriers.

